# Susceptibility to erastin‐induced ferroptosis decreases during maturation in a human oligodendrocyte cell line

**DOI:** 10.1002/2211-5463.12923

**Published:** 2020-07-30

**Authors:** Tomonori Hoshino, Hodaka Yamakado, Ryosuke Takahashi, Shu‐ichi Matsuzawa

**Affiliations:** ^1^ Department of Neurology Graduate School of Medicine Kyoto University Kyoto Japan; ^2^ Present address: Department of Calcified Tissue Biology Graduate School of Biomedical and Health Sciences Hiroshima University Hiroshima Japan

**Keywords:** cell death, ferroptosis, glutathione, MO3.13 cell line, oligodendrocyte

## Abstract

Ferroptosis, a form of iron‐dependent cell death caused by lipid peroxidation, has been implicated in neurological and other disorders. However, the mechanism of ferroptosis in oligodendrocytes is unclear. We tested the susceptibility of MO3.13 cells, an oligodendrocyte line, to ferroptosis after erastin treatment. Immature MO3.13 cells were more susceptible to erastin‐induced ferroptosis than chemically differentiated mature MO3.13 cells. Increased expression of solute carrier family 7 member 11 (SLC7A11), which encodes a cystine/glutamate transporter, and greater glutathione concentrations were observed in mature compared with immature MO3.13 cells, linking glutathione to the resistance of mature MO3.13 cells to erastin‐induced ferroptosis. These findings highlight the usefulness of immature MO3.13 cells in studies of ferroptosis and investigations into neuropathologies that involve oligodendrocytes.

## Graphical abstract

Ferroptosis has been implicated in neurological and other disorders. In this study, we found that immature oligodendrocytes were more susceptible to erastin‐induced ferroptosis than mature oligodendrocytes using human oligodendrocyte cell line MO3.13. These findings highlight the usefulness of immature MO3.13 cells in studies of ferroptosis and investigations into neuropathologies that involve oligodendrocytes.

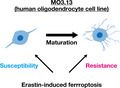

**Abbreviations**
ANOVA: analysis of variance
BSO: buthionine sulfoximine
CNS: central nervous system
EAE: experimental allergic encephalomyelitis
GPX4: glutathione peroxidase 4
GSH: glutathione
MBP: myelin basic protein
MS: multiple sclerosis
MSA: multiple system atrophy
OPCs: oligodendrocyte progenitor cells
PBST: PBS containing 0.3% Triton X‐100
SLC7A11/ xCT: solute carrier family 7 member 11

## Introduction

Ferroptosis is a type of iron‐dependent cell death first reported in 2012 [1]. It has a phenotype that is distinct from other forms of cell death, such as apoptosis and necrosis [1, 2, 3]. Ferroptosis is characterized by the accumulation of lipid peroxidation products and can be induced by chemical compounds, such as erastin and RSL3 [4]. Ferrostatin‐1, liproxstatin‐1, vitamin E, and iron chelators, such as deferoxamine (DFO), have been identified as suppressors of ferroptosis [4]. Although the exact mechanism underlying ferroptosis is unclear, solute carrier family 7 member 11 (SLC7A11; system x_c_ (xCT)) and glutathione peroxidase 4 (GPX4) are some of the key players in ferroptosis [1, 3]. SLC7A11 is the cystine/glutamate antiporter that imports cystine into the cytosol [4]. Erastin irreversibly inhibits SLC7A11 activity, with consequent depletion of the intracellular pool of cysteine, which is a metabolite of cystine and a known precursor of glutathione synthesis [4]. Glutathione depletion has been shown to be sufficient for erastin‐induced ferroptosis, and similar effects have been observed using buthionine sulfoximine (BSO), an inhibitor of γ‐glutamylcysteine synthetase [4, 5]. Moreover, glutathione depletion causes loss of GPX4 activity, resulting in lipid hydroperoxide accumulation and the induction of ferroptosis [4]. Indeed, GPX4 is also directly inhibited by RSL3, which in turn induces ferroptosis [4, 5, 6].

Ferroptosis has been implicated in various diseases, including neurodegenerative disorders, such as Parkinson’s disease (PD). Of the several types of cells in the central nervous system (CNS), including neurons, astrocytes, and microglia, oligodendrocytes are responsible for forming the myelin sheath around neuronal axons that supports neurotransmission. Some types of oligodendrocytes contact neurons without forming myelin sheaths and are involved in supporting neuronal metabolism. Oligodendrocyte progenitor cells (OPCs) are precursors to oligodendrocytes, can proliferate, and are present in the adult CNS. When oligodendrocytes are damaged, OPCs proliferate and differentiate into oligodendrocytes and express oligodendrocyte markers, such as myelin basic protein (MBP) and glycolipid antigen. In the adult brain, OPCs and oligodendrocytes have a 20‐fold higher iron content than astrocytes cultured *in vitro* [7]. One reason for the high iron content in oligodendrocytes is the requirement for iron as a cofactor for myelin synthesis [8]. However, despite its essential role in cells, excessive iron levels induce oxidative stress. Several studies have reported that oligodendrocytes are susceptible to oxidative stress and possibly an overload of iron that would lead to ferroptosis [7, 9, 10, 11].

The accumulation of lipid peroxidation products has been observed in patients with neurological disorders that involve oligodendrocytes, such as multiple system atrophy (MSA) and multiple sclerosis (MS), as well as a mouse model of neuroinflammation, and experimental allergic encephalomyelitis (EAE) [12, 13, 14]; ferroptosis may thus be implicated in these disorders. However, the role of ferroptosis has not been examined in detail. Therefore, in this study, we evaluated erastin‐induced ferroptosis in immature and mature oligodendrocytes using a human oligodendrocyte cell line, MO3.13 cells.

## Materials and methods

### Antibodies

The following primary antibodies were used for immunocytochemistry at the indicated dilutions: mouse anti‐O1 (1 : 500; R&D systems, Minneapolis, MN, USA, cat# MAB1327, RRID:AB_357618), mouse anti‐O4 (1 : 500; R&D systems, cat# MAB1326, RRID:AB_357617), and rabbit anti‐SLC7A11 (1 : 100; ProteinTech, Chicago, IL, USA, cat# 26864‐1‐AP). The secondary antibodies used were as follows: anti‐mouse Alexa Fluor 488 (1 : 1000; Thermo Fisher Scientific, Waltham, MA, USA, cat# A21202, RRID: AB_141607) and anti‐rabbit Alexa Fluor 647 (1 : 1000; Thermo Fisher Scientific, cat# A31573, AB_2536183).

### Cell culture

Culture conditions for immature cells—the human oligodendrocyte cell line, MO3.13 (RRID: CVCL_D357), was cultured for 48–72 h in high glucose Dulbecco's modified Eagle's medium (DMEM) (Wako, Osaka, Japan, cat# 043‐30085) with 10% FBS (Gibco, Grand Island, NY, USA) and a mixture of 100 U/ml penicillin and 100 µg/ml streptomycin (Nacalai Tesque, Kyoto, Japan, cat# 26253‐84) in a humidified incubator with 5% CO_2_ at 37 °C.

For cell maturation studies, 16–24 h after seeding cells, immaturation culture medium was replaced with DMEM containing 0% FBS, 100 U/ml penicillin, 100 µg mL^−1^ streptomycin, and 100 nm phorbol 12‐myristate 13‐acetate (PMA; Sigma‐Aldrich, St. Louis, MO, USA, cat# P1585). Cells were matured for 3 to 7 days with a change in medium every other day.

Prior to treatment, cells were seeded in collagen‐coated 96‐well plates (2000 cells/well) or 60‐mm dishes (100 000 cells/dish) and incubated for 16–24 h.

### Cell viability assay

The viability of MO3.13 cells was measured using a WST‐8 colorimetric assay (Nacalai Tesque, cat# 07553‐44), as per the manufacturer’s protocol. The absorbance of formazan dye, the product of WST‐8 reduction, was measured at 450 nm using ARVO X5 or ARVO X3 (PerkinElmer, Waltham, MA, USA).

### RNA extraction and RT–qPCR analyses

Total RNA was extracted using the RNeasy Plus Mini Kit (Qiagen, Germantown, MD, USA) and reconstituted in RNase‐free water according to the manufacturer's instructions. cDNA was synthesized from total RNA using PrimeScript RT Master Mix (Perfect Real Time; TaKaRa Bio Inc., Shiga, Japan). RT‐qPCR analysis was performed using the LightCycler 480 (Roche, Basel, Switzerland) with LightCycler 480 SYBR Green I Master Mix (Roche). Expression levels for all transcripts were normalized to those of *RPL27*. All primers were obtained from PrimerBank (https://pga.mgh.harvard.edu/primerbank/). The primer sequences used for this study were as follows: human *MBP* (forward) 5ʹ‐GGCCGGACCCAAGATGAAAA‐3ʹ, (reverse) 5ʹ‐CCCCAGCTAAATCTGCTCAGG‐3ʹ; human *SLC7A11* (forward) 5ʹ‐TCTCCAAAGGAGGTTACCTGC‐3ʹ, (reverse) 5ʹ‐AGACTCCCCTCAGTAAAGTGAC‐3ʹ; human *GPX4* (forward) 5ʹ‐GAGGCAAGACCGAAGTAAACTAC‐3ʹ, (reverse) 5ʹ‐CCGAACTGGTTACACGGGAA‐3ʹ; and human *RPL27* (forward) 5ʹ‐TGGCTGGAATTGACCGCTAC‐3ʹ, (reverse) 5ʹ‐CCTTGTGGGCATTAGGTGATTG‐3ʹ.

### Immunocytochemistry

Cells cultured in collagen‐coated 96‐well plates were fixed in 4% paraformaldehyde (PFA) for 20 min, washed twice in PBS for 5 min, and permeabilized in PBS containing 0.3% Triton X‐100 (PBST) for 5 min, before a 15‐min incubation in Blocking One (Nacalai Tesque). Thereafter, cells were washed and then incubated overnight (~16 h) at 4 °C with primary antibodies diluted in PBST containing 5% Blocking One. Next, cells were washed three times with PBST and incubated at room temperature (~25 °C) for 1 h with secondary antibodies. After thorough washing with PBST followed by washing with PBS, cells were visualized for fluorescence using BZ‐9000 or BZ‐X710 (Keyence, Osaka, Japan). At least three independent experiments were performed.

### Thioltracker violet assay

Glutathione (GSH) levels were measured using ThiolTracker Violet (Thermo Fisher Scientific, cat# T10095) according to the manufacturer’s instructions. Briefly, cells were washed twice with prewarmed PBS containing calcium and magnesium (PBS‐C/M), covered with 10 or 20 µm ThiolTracker Violet diluted with prewarmed PBS‐C/M, and incubated in a humidified incubator with 5% CO_2_ at 37 °C for 30 min. Prior to imaging, cells were fixed in 4% PFA prepared with PBS for 20 min. For image capture, cells were rinsed twice with PBS and then counterstained for 5–30 min with 0.5 µg mL^−1^ DAPI. Images were captured using BZ‐9000 (Keyence). For flow cytometry, cells were analyzed using LSRFortessa flow cytometer (BD, Franklin Lakes, NJ, USA), and data were analyzed using FlowJo software version 10.6.1 (BD). At least three biologically independent experiments were performed.

### siRNA treatment

Seeded cells were incubated for 16–24 h and transfected with siRNA, using Lipofectamine RNAiMAX reagent (Thermo), according to the manufacturer’s instructions. After 4‐ to 24‐h incubation, the medium was replaced with fresh medium, and the cells were cultured for further 72 h. The medium was replaced every 48 h. We used Stealth siRNAs (Thermo, cat# 79206969) to knock down SLC7A11 (siSLC7A11; Assay ID: HSS119128). Stealth RNAi Negative Control Medium GC Duplex (Thermo, cat# 46‐2001) was used as the control siRNA (siControl).

### Statistical analysis

All data are presented as mean ± standard error of the mean (SEM). Statistical analyses were performed using R (Version 3.5.1). We used Student's t‐tests for comparisons between two groups and one‐way analysis of variance (ANOVA) followed by Tukey’s multiple comparison test for multiple group comparisons; *P* < 0.05 was considered to indicate statistically significant differences between groups.

## Results

### Erastin‐induced ferroptosis in MO3.13 cells

HT 1080, a human fibrosarcoma cell line, has been extensively used in *in vitro* studies of ferroptosis because it exhibits high sensitivity to erastin, an inducer of ferroptosis [1, 15, 16]. Although erastin‐induced ferroptosis is observed in undifferentiated SH‐SY5Y cells (a human neuroblastoma cell line) and differentiated LUHMES cells (an immortalized dopaminergic neuronal cell line) [17, 18], the mechanism of action of erastin in oligodendrocytes is unclear.

**Fig. 1:**
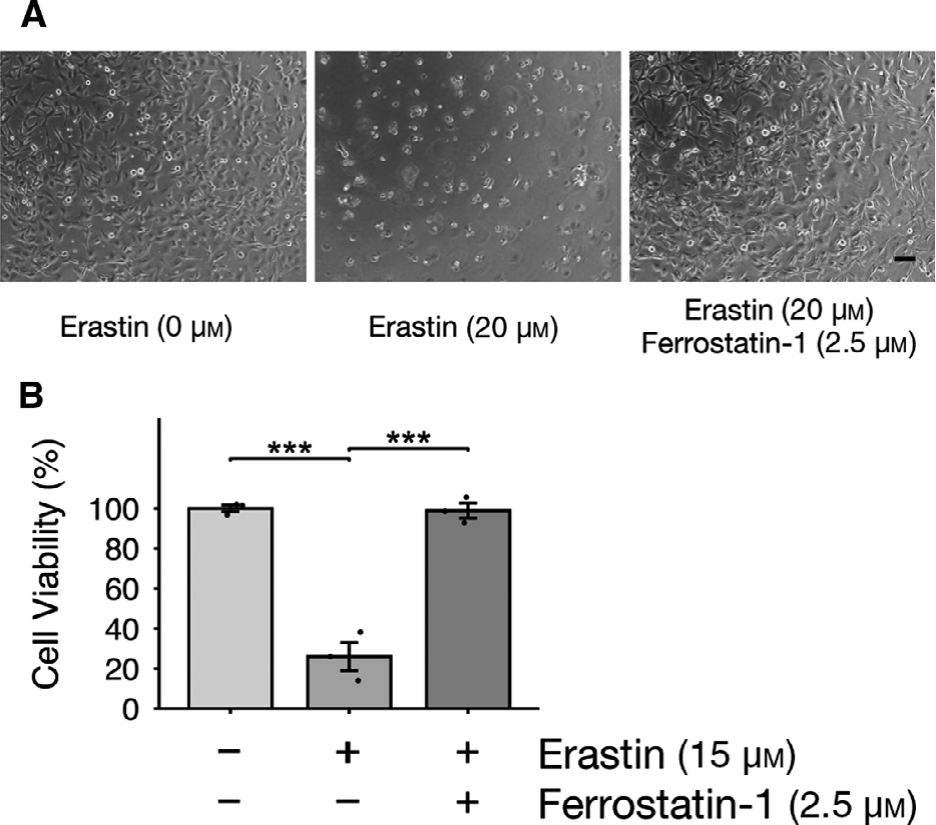
Ferroptosis, observed in erastin‐treated MO3.13 cells, is suppressed by a ferroptosis inhibitor. (A and B) MO3.13 cells were treated with erastin with or without ferrostatin‐1 (a ferroptosis inhibitor) for 24 h. (A) Representative images of immature MO3.13 cells. Scale bar, 100 µm. (B) Cell viability was measured using a WST‐8 assay; three biologically independent samples (n = 3) per group. Data are presented as means ± SEM. ***P < 0.001. One‐way ANOVA followed by Tukey’s *post hoc* test.

To examine the effects of erastin on oligodendrocytes, we used the human oligodendrocyte cell line MO3.13. The immortalized hybrid MO3.13 cells, which were created by the fusion of human oligodendrocytes with a 6‐thioguanine‐resistant human rhabdo‐myosarcoma mutant, have a phenotype characteristic of human oligodendrocytes. The number of MO3.13 cells was significantly decreased following treatment with erastin, as erastin induces ferroptosis (Fig. 1A,B). We confirmed this result by suppressing the erastin‐induced cell death in MO3.13 cells using a ferroptosis inhibitor, ferrostatin‐1 (Fig. 1A,B).

### Mature MO3.13 cells are resistant to erastin‐induced ferroptosis

**Fig. 2:**
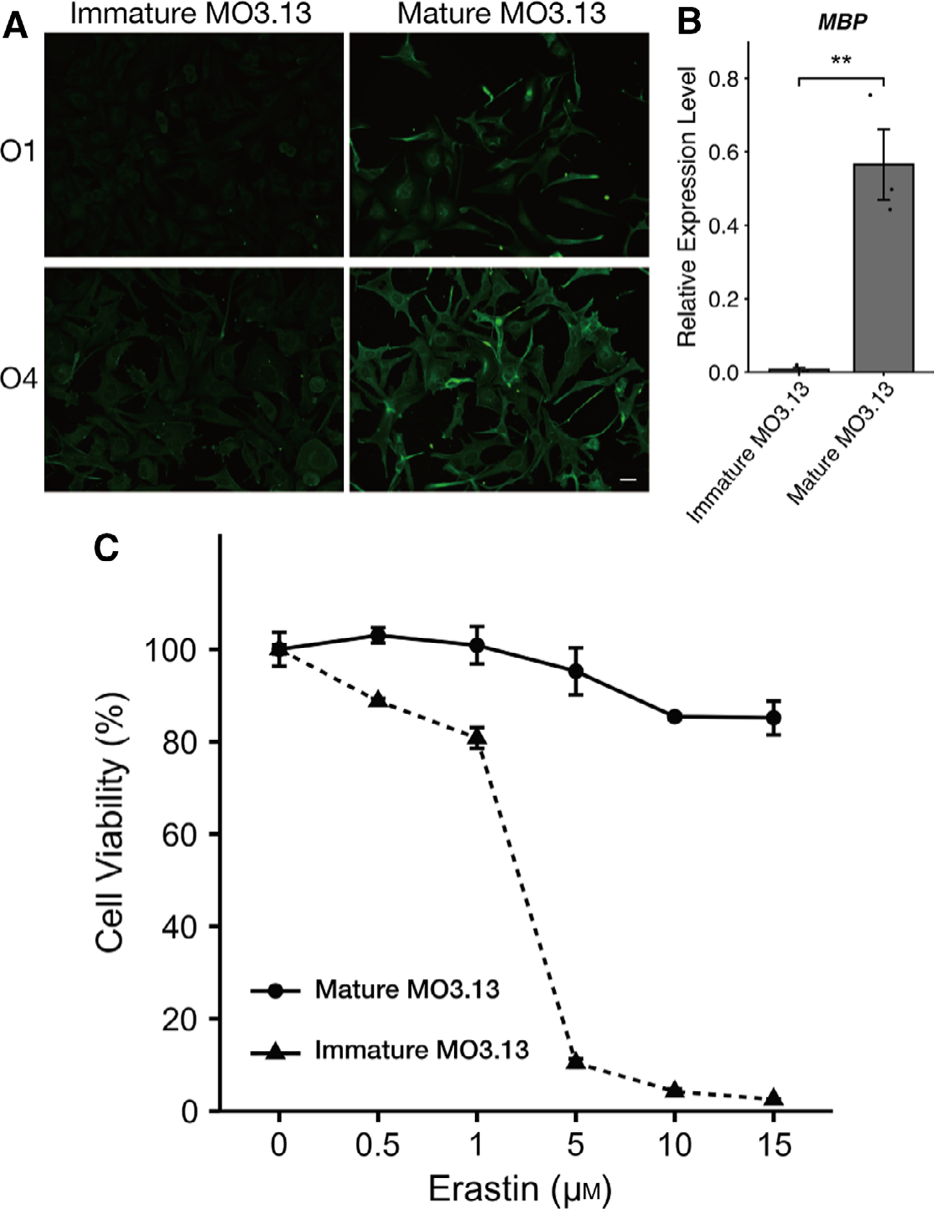
Mature MO3.13 cells are resistant to ferroptosis induced by erastin. (A) Representative images of immunofluorescence staining for oligodendrocyte maturation markers O1 and O4 in MO3.13 cells. Scale bars: 40 µm. (B) RT–qPCR analysis of the expression of *MBP* (oligodendrocyte marker) in immature and mature MO3.13 cells. *MBP* expression levels were normalized to those of *RPL27*; three biologically independent samples (*n* = 3) per group. (C) Immature and mature MO3.13 cells were treated with erastin for 24 h. Cell viability was measured using a WST‐8 assay; three biologically independent samples (*n* = 3) per group. Data are presented as means ± SEM. **P < 0.05 using Student's *t*‐test.

Since MO3.13 cells can be differentiated into mature oligodendrocytes using PMA (a well‐known protein kinase C activator) in the absence of FBS [19], we next investigated whether ferroptosis could be induced by erastin in mature MO3.13 cells. As shown in Fig. 2A,B, we confirmed that high expression levels of maturation markers, such as O1, O4, and MBP, could be observed in mature MO3.13 cells. Intriguingly, mature MO3.13 cells were resistant to ferroptosis, while immature MO3.13 cells remained susceptible to ferroptosis induced by erastin (Fig. 2C).

### Increased SLC7A11 expression in mature MO3.13 cells

**Fig. 3:**
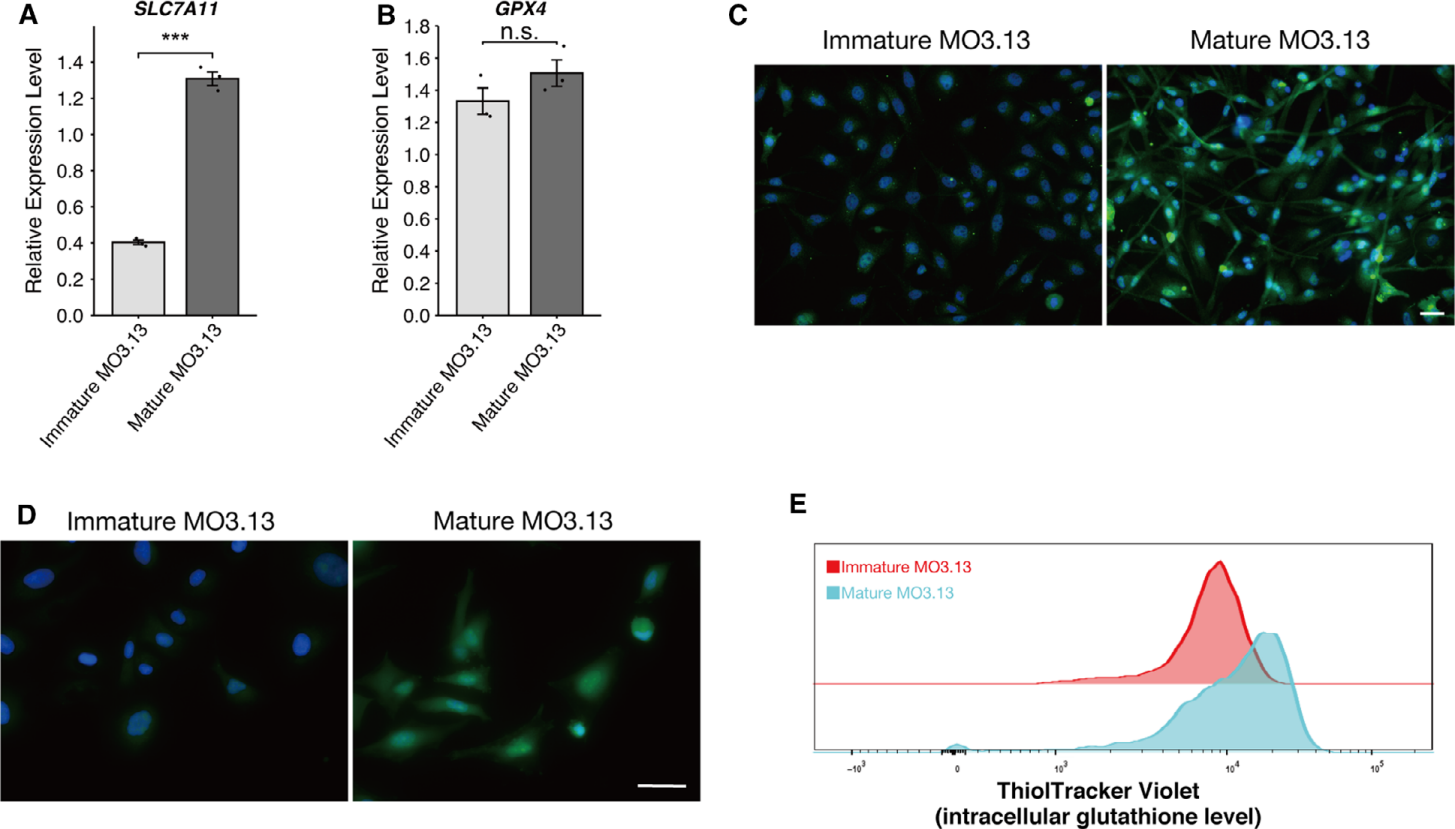
Increased expression levels of SLC7A11 and GSH in mature MO3.13 cells. RT–qPCR analysis of the expression of ferroptosis markers *SLC7A11* (A) and *GPX4* (B) in immature and mature MO3.13 cells. Gene expression levels were normalized to those of *RPL27*; three biologically independent samples (*n* = 3) per group. Data are presented as means ± SEM. n.s., not significant. ***P < 0.01 by Student's *t*‐test. (C) Representative images of immunofluorescence staining for SLC7A11 (green) counterstained with DAPI (blue) in MO3.13 cells. Scale bars: 50 µm. (D) Representative images of immature and mature MO3.13 cells stained with ThiolTracker Violet (green) counterstained with DAPI (blue). Scale bars: 50 µm. (E) Flow cytometry analysis of ThiolTracker Violet in immature and mature MO3.13 cells. At least three biologically independent experiments were performed.

Erastin inhibits SLC7A11 activity, which can then promote ferroptosis because of the depletion of glutathione [1]. To examine the mechanisms underlying the high ferroptosis resistance in mature MO3.13 cells, we evaluated the expression of ferroptosis‐related genes in both immature and mature MO3.13 cells using RT–qPCR and immunocytochemistry. Higher expression levels of SLC7A11 could be observed in mature MO3.13 cells compared with immature cells (Fig. 3A,C). In contrast, the expression of *GPX4* was not significantly different between immature and mature MO3.13 cells (Fig. 3B). SLC7A11 is required for glutathione (GSH) synthesis and contributes to protective effects against oxidative stress [1, 4, 20, 21]. Therefore, we measured GSH levels using an intracellular thiol probe, ThiolTracker Violet, which detects reduced forms of GSH. We detected higher levels of GSH in mature MO3.13 cells compared with those in immature MO3.13 cells (Fig. 3D,E).

**Fig. 4:**
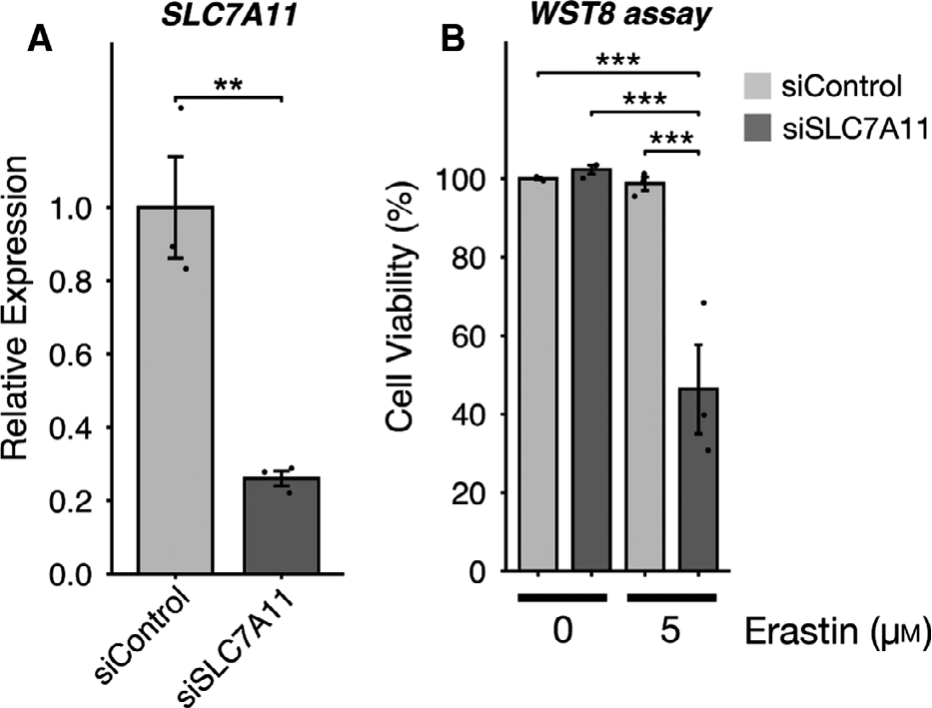
Knockdown of SLC7A11 get susceptibility to erastin‐induced ferroptosis in mature MO3.13 cells. (A) RT–qPCR analysis of the expression of ferroptosis marker *SLC7A11* in mature MO3.13 cells. Gene expression levels were normalized to those of *RPL27*; three biologically independent samples (*n* = 3) per group. Data are presented as means ± SEM. **P < 0.01 and ***P < 0.001 by Student's *t*‐test. (B) Cell viability was measured using a WST‐8 assay; three biologically independent samples (*n* = 3) per group. Data are presented as means ± SEM. ***P < 0.001. One‐way ANOVA followed by Tukey’s *post hoc* test.

Next, to ascertain whether the tolerance of mature MO3.13 cells to erastin‐induced ferroptosis is mediated by SLC7A11, we conducted siRNA experiments to knock down SLC7A11. As shown in Fig. 4A, the result of RT–qPCR showed that SLC7A11 expression was reduced by 75% in MO3.13 cells, 72 h after the siRNA treatment. Using siRNA interference, we observed that SLC7A11 knockdown alters the tolerance of mature MO3.13 cells to erastin‐induced ferroptosis in the absence of SLC7A11 (Fig. 4B). Furthermore, we found that SLC7A11 knockdown increased vulnerability of mature MO3.13 cells to ferroptosis. In summary, we found that mature MO3.13 cells were resistant to erastin‐induced ferroptosis, probably as a consequence of upregulated SLC7A11 expression and a subsequent increase in GSH levels.

## Discussion

In this study, we demonstrated that erastin‐induced ferroptosis occurs in an immature human oligodendrocyte cell line, MO3.13 cells. When these cells were matured through chemical‐induced differentiation, they became resistant to erastin‐induced ferroptosis by upregulating SLC7A11 expression and increasing GSH synthesis. To the best of our knowledge, this is the first *in vitro* study of ferroptosis in oligodendrocytes.

Ferroptosis is a lipid peroxidation‐dependent mechanism of cell death. The accumulation of lipid peroxidation products in neurological disorders that show an involvement of oligodendrocyte cell death, such as MSA and MS, is suggestive of the involvement of ferroptosis [12, 14]. In primary neurons, the lipid peroxidation product 4‐hydroxynonenal (4‐HNE) triggers the secretion of extracellular vesicles that contain oligomeric α‐synuclein species, which are related to PD and MSA pathology [22]. This suggests that inhibiting lipid peroxidation associated with ferroptosis may have therapeutic potential.

We induced ferroptosis in immature MO3.13 cells, and this process was inhibited by ferrostatin‐1 treatment. This finding indicates that immature MO3.13 cells represent a useful model to study ferroptosis in oligodendrocytes. Surprisingly, we found that mature MO3.13 cells are resistant to ferroptosis. This unexpected result may be explained by the fact that expression levels of SLC7A11 and GSH were higher in mature MO3.13 cells compared with those in immature MO3.13 cells. Consistent with our results, primary OPCs have been reported to be more susceptible to cystine depletion than primary oligodendrocytes in rats [9]. Similar results were observed in the mature MO3.13 cells that were differentiated in the absence of FBS without protein PKC activator PMA (data not shown), indicating that resistance to ferroptosis in mature MO3.13 cells does not occur via protein kinase C activation.

Ferroptosis has been implicated in many pathological conditions, including various cancers and age‐related neurological disorders, such as Alzheimer’s disease and PD [4, 14, 23, 24, 25, 26]. Oligodendrocyte dysfunction can also be observed in these neurological disorders [27, 28]. Considering that iron accumulation increases with aging and lipid peroxidation agents have protective effects in these diseases [29, 30], detailed studies on ferroptosis in OPCs and oligodendrocytes are warranted. A limitation of this study is that it only examined a human oligodendrocyte cell line. Primary OPCs/oligodendrocytes and *in vivo* models would be ideal for future investigations of ferroptosis. In conclusion, our findings show that immature MO3.13 cells represent a useful model for studies of ferroptosis and the discovery of therapeutic targets in neurological disorders that involve oligodendrocytes.

## Conflict of interest

The authors declare no conflict of interest.

## Author contribution

TH and SM designed the experiments. TH performed the experiments. SM, HY, and RT critically discussed the study design and interpreted the results. TH, SM, HY, and RT wrote the manuscript. All authors reviewed and approved the final version of the manuscript.

## Data Availability

The raw data of this study are available from the corresponding author on reasonable request.
